# Exploring the Ability of ChatGPT to Act as a Research Aid in Otolaryngology

**DOI:** 10.1007/s12070-024-05101-z

**Published:** 2024-10-01

**Authors:** Ariana L. Shaari, Isabel Herzog, Emily Kokush, Sylvia Zabielski, Sydney Langer, Andrey Filimonov

**Affiliations:** 1 Department of Otolaryngology- Head and Neck Surgery, Rutgers New Jersey Medical School, Newark, New Jersey USA; 2 Johns Hopkins University, Baltimore, Maryland USA

**Keywords:** Artificial intelligence, Otolaryngology, Surgical education

## Abstract

Recently artificial intelligence (AI) platforms have developed at a rapid pace. To date no studies have explored AI platform ChatGPT’s ability to serve as an aid in research in the field of otolaryngology. The objective of our study is to evaluate the ability of ChatGPT to generate unique research ideas relevant to Otolaryngology. ChatGPT was tasked to generate novel research project ideas. Seven categories for all otolaryngology subspecialties were created: general otolaryngology, facial plastics and reconstructive surgery, rhinology and skull base surgery, pediatrics, head and neck oncology, laryngology, and otology/neurotology. Within each of the subspecialties, ChatGPT was prompted to provide research ideas for two specific research topics in order to gauge ChatGPT’s ability to explore specific domains. Ten prompts were entered for general otolaryngology, and five prompts were entered for each subspecialty and subspecialty topic. ChatGPT was subsequently prompted with the same query to generate systematic review ideas for each category. Ideas were deemed novel if there were no similar systematic reviews retrieved during the literature review on PubMed, Scopus, or Web of Science. Ideas were graded for Clinical Relevance on a scale of 0 to 5, 5 being considered highly relevant/would contribute greatly to patient care and 0 being considered not relevant/not beneficial to patient care. Two reviewers graded each response. Out of 100 systematic review ideas generated by ChatGPT, we found that the artificial intelligence platform was incapable of creating unique systematic review research ideas. However, the ideas that it did generate were largely feasible and clinically relevant. Future studies should investigate the ability of ChatGPT to generate research inquiries with non-systematic review methodologies and in more specific otolaryngology topics.

## Introduction

Artificial intelligence platforms have demonstrated remarkable progression in recent years. ChatGPT is a large-language model that was released by San Francisco based company OpenAI. Since being made available to the public in November 2022, ChatGPT has become the fastest-growing application in history [1, 2]. The potential of ChatGPT in medicine is vast and has already been explored in studies evaluating its ability to pass licensing examinations [3], provide post-operative instructions following otolaryngologic surgery [4], and respond to patient questions about common otolaryngology conditions [5]. Early investigations have been conducted into the ability of ChatGPT to assist in research. Gupta et al. found that ChatGPT is an exceptional instrument in generating new research ideas in the plastic surgery realm [6]. To our knowledge, no studies have investigated the ability of ChatGPT to generate systematic review ideas within otolaryngology. We sought to determine if ChatGPT is capable of generating novel systematic review ideas and if so, are the ideas both clinically relevant and feasible for otolaryngologists (Table 1).

**Table 1:** Number of total and Unique Research Ideas Generated by ChatGPT for General Otolaryngology and each subspeciality

Category	Subcategory	Total Number of Prompts	Total Number of Novel Ideas	Percent of Novel Ideas	% novel General vs. Specific Topics	No/% of Novel Ideas (Subspecialty Totals)
General Otolaryngology (*n* = 10)	N/A	10	2	20	20	2 (20%)
Facial Plastic and Reconstructive Surgery (*n* = 15)	General	5	3	60	60	7 (47%)
	Septoplasty	5	2	40	40	
	Rhytidectomy	5	2	40		
Head and Neck Surgical Oncology (*n* = 15)	General	5	2	40	40	3 (20%)
	Thyroid cancer	5	0	0	20	
	TORS	5	1	20		
Laryngology (*n* = 15)	General	5	1	20	20	1 (7%)
	Recurrent respiratory papillomatosis	5	0	0	0	
	Laryngeal Cancer	5	0	0		
Otology & Neurotology (*n* = 15)	General	5	0	0	0	2 (13%)
	Cochlear Implantation	5	0	0	20	
	Cholesteatoma	5	2	40		
Rhinology, Allergy and Skull Base Surgery (*n* = 15)	General	5	2	40	40	6 (40%)
	Frontal Sinus Fractures	5	3	60	40	
	Allergic Rhinitis	5	1	20		
Pediatric Otolaryngology (*n* = 15)	General	5	2	40	40	6 (40%)
	Tympanostomy Tubes	5	2	40	40	
	Laryngomalacia	5	2	40		

## Methods

**Fig. 1:**
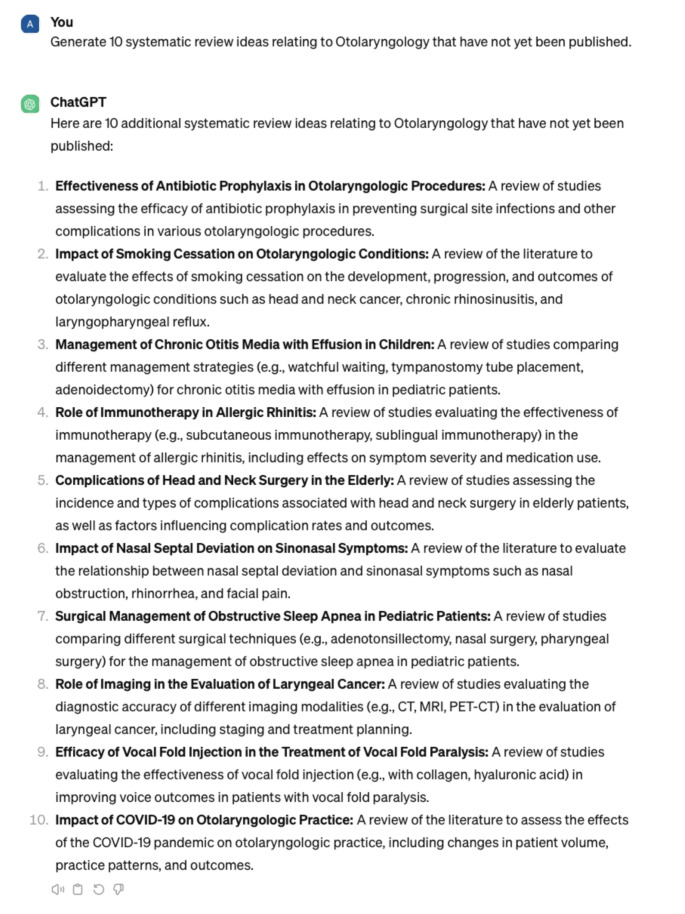
Sample prompt from ChatGPT regarding general otolaryngology project ideas

**Fig. 2:**
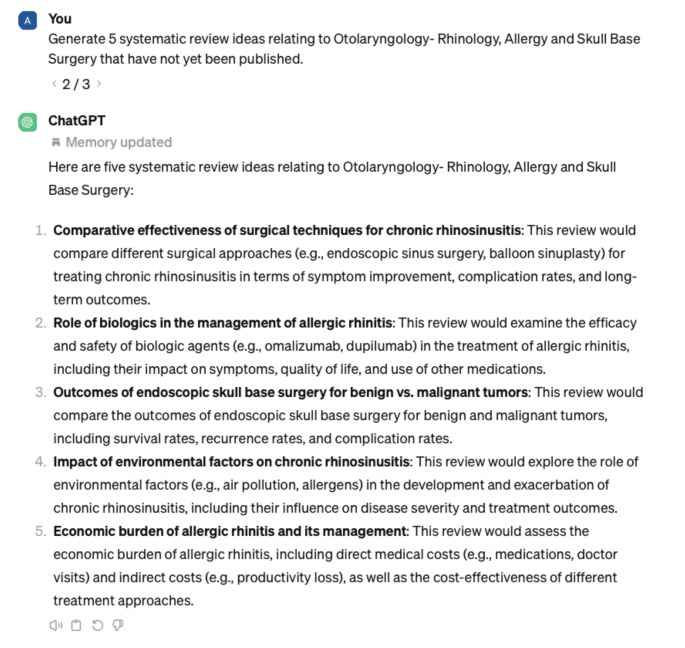
Sample prompt from ChatGPT regarding otolaryngology subspecialty project ideas

**Fig. 3:**
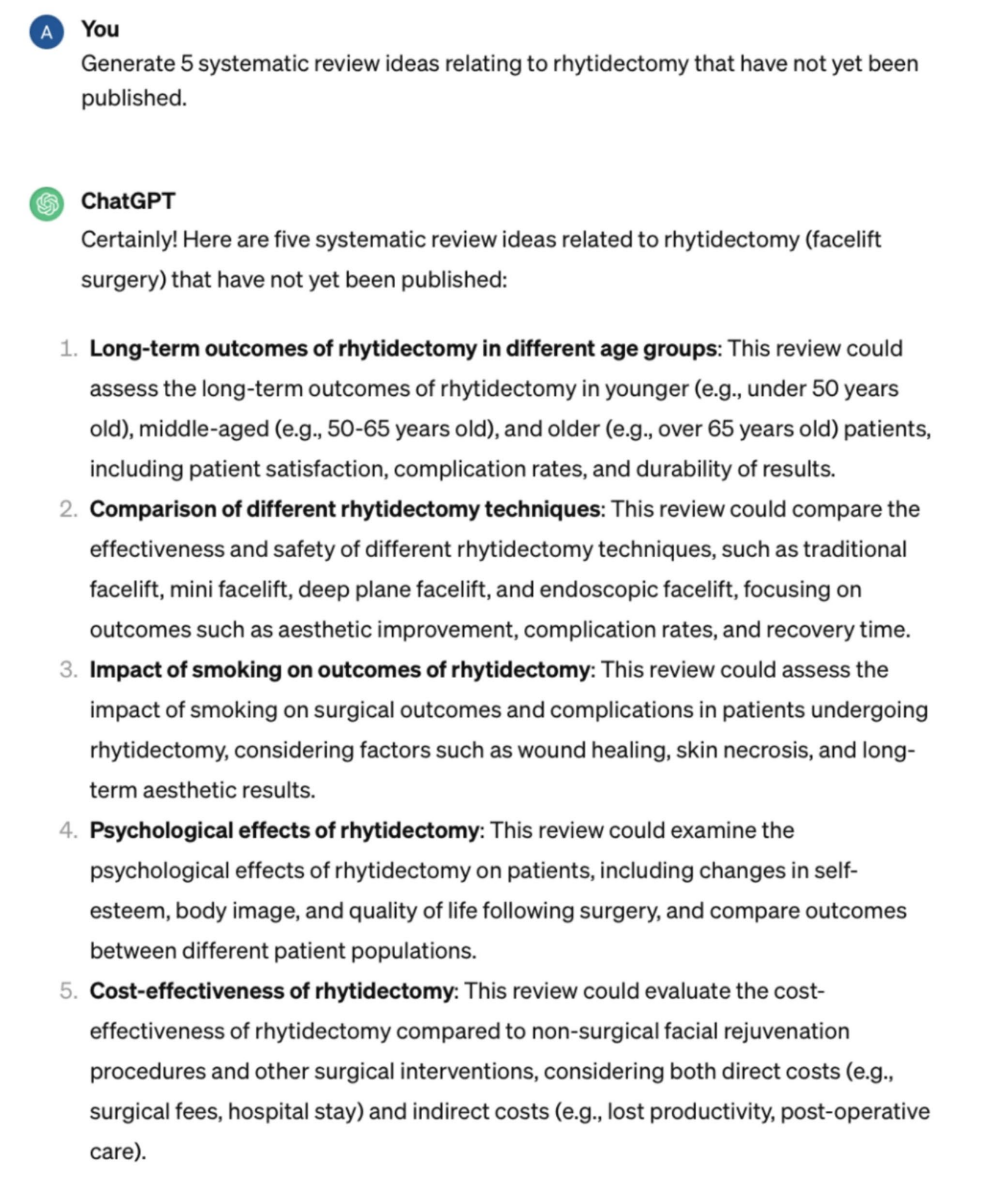
Sample prompt from ChatGPT regarding specific otolaryngology project ideas

This investigation review met criteria for nonhuman subject research and was therefore exempt from IRB review. We tasked ChatGPT with thinking of novel research project ideas loosely following the methods of previous studies [7]. Seven categories for all subspecialties were created: general otolaryngology, facial plastics and reconstructive surgery, rhinology and skull base surgery, pediatrics, head and neck oncology, laryngology, and otology/neurotology. Within each of the subspecialties, ChatGPT was prompted to provide research ideas for two specific research topics in order to gauge ChatGPT’s ability to explore specific domains. Ten prompts were entered for general otolaryngology, and five prompts were entered for each subspecialty and subspecialty topic (Fig. 1–3).

ChatGPT was subsequently prompted with the same query to generate systematic review ideas for each category. For example, for the facial plastics and reconstructive surgery category, ChatGPT was asked “ Generate 5 novel systematic review ideas about otolaryngology- facial plastics and reconstructive surgery that have not yet been published”; and for the topic of “septoplasty” within facial plastics, the prompt was,”Generate 5 systematic review ideas relating to septoplasty that have not yet been published. ”

Ideas were deemed novel if there were no similar systematic reviews retrieved during the literature review on PubMed, Scopus, or Web of Science. Ideas were graded for Clinical Relevance on a scale of 0 to 5, 5 being considered highly relevant/would contribute greatly to patient care and 0 being considered not relevant/not beneficial to patient care. Two reviewers graded each response. A third reviewer was available if disagreements arose. Feasibility was deemed via “Yes” or “No” response.

## Results

Of the 100 systematic review ideas that ChatGPT generated, a total of 27% of ideas were considered novel (*n* = 27), meaning they had not been already published, and 73% were non-novel (Table 1). In terms of subspecialty, ChatGPT generated the most novel ideas for facial plastic and reconstructive surgery (47%), followed by rhinology/allergy/skull base (40%) and pediatric otolaryngology (40%). For specific subspecialty topics, the percent of novel ideas ranged from 0 to 60%, with the most novel subspecialty ideas in facial plastic and reconstructive surgery, rhinology allergy/skull base, and pediatrics. Of the 27 novel ideas, 55% (*n* = 15) were about specific topics in a subspecialty and the remaining 44% (*n* = 12) were general topics relating to subspeciality. Of the 27 ideas that were novel, ChatGPT ideas had a Clinical Relevance of 4.07 out of 5, and 100% were considered feasible.

## Discussion

ChatGPT is revolutionary for its capabilities to interact with human users in a life-like manner and for its rapid spread across the public. Per February 2023, over 100 million people utilize ChatGPT [1]. In our investigation, we sought to determine if ChatGPT is a reliable aid in the generation of systematic review research ideas within otolaryngology. Overall we found that ChatGPT generated less than 30% of novel ideas. This disparity existed across all subspecialties and for specific topics. Notably however, of the ideas that were novel, ChatGPT was highly successful in generating both clinically relevant and feasible ideas.

Our findings both contrast with and support the limited published literature evaluating ChatGPT’s ability to serve as a clinical research aid. Gupta et al. found that ChatGPT had a 65% accuracy rate in generating novel ideas within the field of cosmetic plastic surgery [7]. When dissecting topics further by subspecialty, they found that for specific topics such as rhinoplasty and blepharoplasty, ChatGPT had a 50-80% accuracy rate in generating new ideas [7]. Further research corroborates that ChatGPT was more accurate for specific ideas [6]. Although we found that ChatGPT generated significantly fewer novel ideas (27%) than previous studies, our results supported Gupta et. al.’s findings. in that ChatGPT was more accurate in generating novel ideas for specific topics. Potential explanations for our observations could be attributed to the data sets on which ChatGPT is trained on and the version of ChatGPT utilized [8]. Additionally, the relatively few novel ideas generated by ChatGPT in our study align with Nachalon et. al.’s work that demonstrated similar findings with prompts related to dysphagia [9].

## Conclusion

ChatGPT was occasionally able to generate novel systematic review ideas within the field of otolaryngology. The novel ideas that it generated were highly clinically relevant and feasible. Researchers and clinicians should be aware of the limitations of this technology when utilizing it as a research aid. Further studies should investigate the ability of ChatGPT to develop more specific project ideas as the platform evolves.
